# Dietary habits and metabolic response improve in obese children whose mothers received an intervention to promote healthy eating: randomized clinical trial

**DOI:** 10.1186/s12889-020-09339-4

**Published:** 2020-08-14

**Authors:** Iris Nallely López-Contreras, Jenny Vilchis-Gil, Miguel Klünder-Klünder, Salvador Villalpando-Carrión, Samuel Flores-Huerta

**Affiliations:** 1 Gastroenterology and Nutrition Department, Hospital Infantil de México Federico Gómez, Ministry of Health (SSA), Mexico City, Mexico; 2 Epidemiological Research Unit in Endocrinology and Nutrition, Hospital Infantil de México Federico Gomez, Ministry of Health (SSA), Dr. Márquez No 162, 06720 Mexico City, Mexico; 3 Medicine Faculty, National Autonomous University of Mexico, Mexico City, Mexico; 4 Deputy Director of Research, Hospital Infantil de México Federico Gómez, Ministry of Health (SSA), Mexico City, Mexico; 5 Research Committee, Latin American Society for Pediatric Gastroenterology, Hepatology and Nutrition (LASPGHAN), Mexico City, Mexico

**Keywords:** Feeding behavior, Intervention, Insulin resistance, Dietary habits, Childhood obesity

## Abstract

**Background:**

Lifestyles habits such as eating unhealthy foodscommence at home and are associated with the development of obesity and comorbidities such as insulin resistance, metabolic syndrome, and chronic degenerative diseases, which are the main causes of death in adults. The present study compared changes in dietary habits, behaviors and metabolic profiles of obese children whose mothers attended at the hospital to group sessions, with those who received the usual nutritional consultation.

**Methods:**

Randomized clinical trial, 177 mother/obese child pairs participated, 90 in the intervention group and 87 in the control group. The intervention group attended six group education sessions to promote healthy eating, being this an alternative of change of habits in children with obesity. The control group received the usual nutritional consultation; both groups were followed up for 3 months. Frequency of food consumption, behaviors during feeding in the house and metabolic profile was evaluated. Mixed effect linear regression models were used to evaluate the effect of the intervention on the variables of interest, especially in HOMA-IR.

**Results:**

The intervention group reduced the filling of their dishes (*p* = 0.009), forcing the children to finish meals (*p* = 0.003) and food substitution (*p* <  0.001), moreover increased the consumption of roasted foods (*p* = 0.046), fruits (*p* = 0.002) and vegetables (*p* <  0.001). The children in the control group slightly increased HOMA-IR levels (0.51; 95% CI − 0.48 to 1.50), while the children in the intervention group significantly decreased (− 1.22; 95% CI − 2.28 to − 1.16). The difference in HOMA-IR between the control and intervention group at the end of the follow-up was − 1.67; 95% CI: − 3.11 to − 0.24.

**Conclusions:**

The educational intervention improved some eating habits at home, as well as HOMA-IR levels; why we consider that it can be an extra resource in the management of childhood obesity.

**Trial registration:**

Clinicaltrials.gov, NCT04374292 (Date assigned: May 5, 2020). Retrospectively registered.

## Background

In recent decades, obesity in children and adolescents has been on the rise worldwide [1, 2]. The distribution of overweight and obesity varies widely according to age group, race, region, urbanization and socioeconomic status. Metabolically, obesity is caused by an imbalance in which energy intake exceeds expenditure in a persistent fashion. Low energy expenditure is caused by decreased physical activity and increased sedentary. The energy imbalance of overweight children results from the unhealthy lifestyles acquired at home, and health providers need to inform parents on how to change unhealthy feeding and sedentary habits to preserve the children health [3–5]. People are consuming more energy-intensive industrialized foods and beverages than natural foods and water. Meanwhile, they perform less physical activity and are more sedentary [6, 7]. The National Nutrition Surveys showed that from 1999 to 2018 the prevalence increased from 26.9 to 35.6%, indicating that this problem prevails with unacceptably high figures for this age group [8–10]. Obesity is associated from childhood with comorbidities such as metabolic syndrome, hypertriglyceridemia and type 2 diabetes constituting a major public health problem [10–12]. Therefore, it is important to develop alternative strategies to prevent this health problem since early age. Additionally, obesity decreases children’s self-esteem, thus deteriorating their psychosocial well-being [13]. Parents with obese children turn to health clinics for assistance with this problem; once diagnosed, and after clinical and metabolic procedures, given the shortage of pharmacological resources, they are prescribed an initial diet plan in accordance to their age and gender [14], with mothers receiving instruction on how to prepare and follow this plan at home. An evaluation of this management proven little effectiveness in reducing children’s body weight and has shown elevated dropout rates [15]. When the study was conducted, the Obesity Clinic at the Federico Gomez Children’s Hospital of Mexico had not yet implemented programs to assist families in changing their lifestyles. Interventions involving parents and thatinclude healthy food, physical activity, and behavioral changes components, are accepted as the path with the best results for treating child obesity [16] however, these interventions are complex and costly. The next option is to direct interventions at both parents, considering that they are agents of change in their children’s habits. Results depend on the design and duration of the study, with studies of over a year and more frequent contact leading to better results; but success also depends on the expected outcome variable; success is rare when programs are designed to reduce children’s body mass index [17]. In large cities, interventions directed at both parents face many complications, which leave the option of directing interventions specifically at mothers, who most often take children to the hospital. Also, they are the main agent behind dietary habits in the household [18]. In this context, the aim of this study is to evaluate the change in eating behaviors, metabolic condition, and nutritional status measured by anthropometry, in children with obesity who were prescribed a diet to reduce their body weight in the usual nutritional consultation, in comparison to children whose mothers participated in an intervention of six group sessions to acquire healthy dietary habits.

## Methods

### Design

The Obesity Clinic at Federico Gomez Children’s Hospital of Mexico conducted arandomized clinical trial between January 2011 and December 2014 with approval from the hospital’s Research, Ethics and Biosecurity Committee. This study is reported according to the CONSORT guidelines (Additional file 1). After providing written consent, 177 children with obesity (BMI ≥ 95 pc) of age 5–11 years and their mothers were randomly assigned to participate in the intervention group or the control group. None of the participating children were receiving pharmacological treatment for obesity, were morbidly obese or were associated with a genetic syndrome. Mothers of intervention group (*n* = 90) attended six weekly group sessions, lasting 90 min. Sessions were taught by nutritionists of the clinical nutrition course who rotate in the Obesity Clinic of this hospital and were standardized in the procedures performed.

(See session content in Table 1).

**Table 1:** Topics covered in each session with intervention group mothers

Session	Content
1	Dietary and physical activity habits and their link to obesity and cardio-metabolic diseases. Children learn about healthy eating habits and health risks at home.
2	Food-preparation processes. Selecting and purchasing food and beverages; importance of food groups and their impact on health; importance of fruit and vegetables. Balance between food groups, source of foods, organic or industrialized. Family menu preparation. Eating at the table to be more present during food intake.
3	Habits and behaviors surrounding eating processes identified as health or risk factors, such as energy density, portion-size control, controlling emotional eating.
4	Beverages. Water versus sugar-sweetened drinks prepared at home or purchased at the store.
5	Preventing the risk of cardiovascular diseases by learning healthy eating habits and practicing these habits at home.
6	Integration. Practicing the skills learned during the intervention in each stage of preparing food and eating
	The intervention group did not cover physical activity topics.

The key message was that healthy dietary habits and health risks are acquired at home and that opportunities for change can be identified in the processes that surround mealtimes. It begins with selecting and purchasing food, followed by preparation and consumption behaviors [19]. Mothers were encouraged to participate in the sessions which involved the use of food models, videos, slides, and, in some cases, real food. Upon completing each session, mothers were given printed material to add to a home consultation manual. Children in this group were not prescribed diets to reduce their body weight. Control group mothers and children (*n* = 87) were given the usual nutritional consultation which consists of prescribed diets that covered their energy requirements according to their age and gender [14]. Similarly, control group mother/child pairs received information regarding food groups and portion sizes, were trained in the use of the food equivalence system to encourage variation and were instructed on how to prepare the diet at home. Neither group of children received physical activity programs. Upon concluding consultations and group sessions, mother/child pairs from both groups were asked to return for monthly follow-upsover the next 3 months.

The assignment to intervention or control groups was made using a block randomization with 8 mother/child pairs in each block to assure equal allocation to groups [20]. A study collaborator obtained a computer-generated randomization list using the Stata 11.0 program. Children were randomized at the end of the baseline examination. The calculation of the sample size was estimated using the formula of comparison of proportions in eating habits between the mothers/children dyad of the intervention and control groups [21, 22] to detect a 20% difference in eating habits, with significant level of 5 and 80% power; the size of sample required was 72 mother/children pairs per group and to allow for 20% drop-out during the follow-up, we aimed at recruiting 86 mother/children dyad per group.

### Measurements at the beginning and end of the study

#### Anthropometry

The weight, height and waist circumference of children in both groups were measured according to international procedures. Weight was measured on a mechanical scale (Seca model-700, SECA Corp., Hamburg, Germany) with 50 g precision. Height was measured on a stadiometer (SECA model-225, SECA Corp., Hamburg, Germany) 0.1 cm precision. Meanwhile, waist circumference was measured at the end of an exhalation with non-elastic flexible tape (Seca model-200, SECA Corp., Hamburg, Germany) in a standing position at the midpoint between the lower costal border and the iliac crest. Body Mass Index (BMI) and percentile value were calculated using CDC data for reference [23], children with a BMI ≥ 95 pc were categorized with obesity.

#### Blood pressure

Blood pressure was measured on children’s right arm with a mercury sphygmomanometer (ALPK2, Tokyo, Japan) using a cuff that suited arm length and perimeter and following 2004 National High Blood Pressure Education Program guidelines [24].

#### Questionnaires

A questionnaire of sociodemographic data was applied to the mothers at baseline. In addition, a survey of family feeding habits at home at baseline and 3 months was applied, the mother was asked about the child’s breakfast habit, family feeding habits at the time of sitting at the table (place the salt shaker, sugar and sweetened soft drinks on the table, fill the plate, toask a food additional portion, force the child to finish the meal, accept the exchange of food to what the child wants), frequency of consumption of food (fried, roasted, fruits and vegetables) and drinks (simple water, sugary drinks prepared at home, natural juice, industrialized juice and soft drinks). The amounts of food consumption were not evaluated. A survey of physical activity and sedentary activities was applied [25]; no physical activity intervention was performed during this study.

#### Blood sample

Five mL venous blood samples after 12 h fasting periods were drawn to determine glucose (mg/dL) (hexokinase method Dimension RxL Max, Siemens Euro, DPC, Llanberis, UK), insulin (mIU/mL) (chemiluminescence IMMULITE 1000, Siemens), HDL cholesterol (mg/dl) (enzymatic reaction/catalase, using ADVIA® 1800 equipment), and triglycerides (mg/dl) (ILAB 300, Instrumentation Laboratory, Barcelona Spain). Participating children’s HOMA-IR index was obtained through glucose and insulin data (I_f_xG_f_/22.5) [26].

### Statistical analysis

Central tendency measures were used to describe the study population’s baseline characteristics. Student’s *t*-test for independent samples was used to compare continuous variables with normal distribution or Mann-Whitney test no normal distribution, such as socio-demographic data, dietary habits, feeding behaviors, and biochemical and anthropometric data. Pearson *X*^2^ test was used to compare proportions between groups. The equal proportions test was used to assess the difference in proportions in dietary habits and behaviors between groups. Mixed effect linear regression models were used to evaluate the effect of the intervention on the variables of interest (BMI percentile, waist circumference, C-HDL, triglycerides, glucose, insulin and HOMA-IR) during follow-up. These models were adjusted for baseline data of the dependent variable, time, gender, baseline age and BMI percentile and maternal schooling. A model of mixed-effects linear regression was used to assess the interaction between the groups and the time of evaluation for the dependent variable HOMA-IR. Mean HOMA-IR by group (intervention or control) and by time was calculated and a graphic was done using marginal analysis. Statistical significance was considered at *P* < 0.05. Analyses were carried out with STATA SE v.12.0 (Stata Corp, CollegeStation, TX, USA).

## Results

**Fig. 1:**
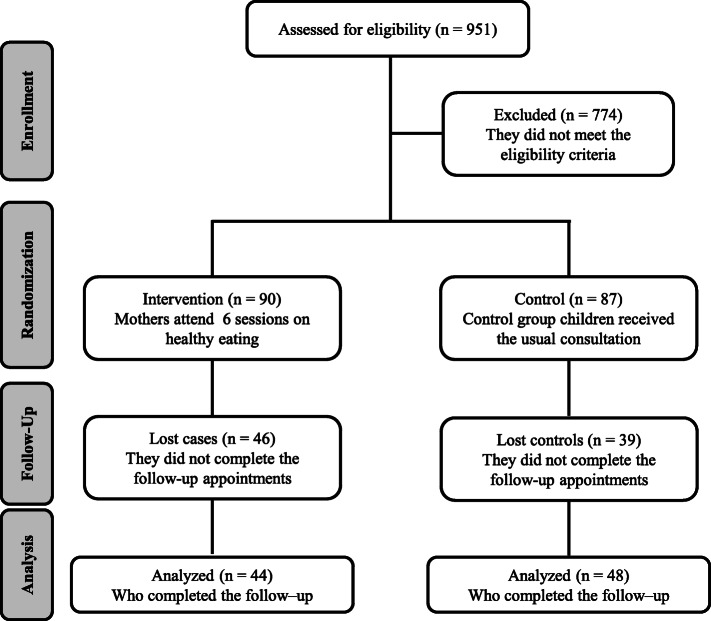
CONSORT flow diagram of participants throughout the study

In the intervention group, 44/90 pairs (49%) completed the follow-up; in the control group 48/87 pairs (55%) completed the follow-up, as shown in Fig. 1.

Table 2 features the children’s baseline anthropometric, clinical, and metabolic characteristics, showing that both groups are comparable. Both groups BMI was >95pc, and both had HOMA-IR indexes of ≥3.4. Regarding physical and sedentary activities, the intervention group children spent 5.5 and 13 h/wk. on each of these activities; control group children spent 7 and 14 h per week on each of these activities, respectively.

**Table 2:** Anthropometric, clinical and metabolic characteristics of participating children, at the start of the study

Characteristics	Intervention (*n* = 90) Mean ± SD	Control (*n* = 87) Mean ± SD	*p*^b^
Age (y)	8.6 ± 1.5	8.7 ± 1.3	0.356
Male (%)	51 (56.7)	50 (57.5)	0.914
**Anthropometrics**			
Weight (kg)	49.1 ± 12.5	48.5 ± 10.3	0.783
Height (cm)	138.5 ± 10.3	137.9 ± 9.4	0.661
BMI (kg/m^2^)	25.0 ± 3.4	25.2 ± 3.3	0.673
BMI (percentile)	97.5 ± 2.3	97.6 ± 2.1	0.937
Waist circumference (cm)	82.9 ± 10.1	82.4 ± 9.0	0.700
**Blood Pressure**			
Systolic (mmHg)	92.6 ± 9.4	93.7 ± 9.9	0.392
Systolic (Percentile)^a^	17.4 (5.9–30.2)	19.6 (7.3–33.1)	0.580
Diastolic (mmHg)	59.1 ± 8.2	60.8 ± 10.7	0.190
Diastolic (Percentile)^a^	45.5 (25.4–56.6)	45.4 (25.3–78.0)	0.470
**Metabolics**			
Glucose (mg/dL)	88.8 ± 7.9	89.1 ± 16.1	0.934
Cholesterol HDL (mg/dL)^a^	45 (34–53)	43 (35.7–52)	0.961
Triglycerides (mg/dL)^a^	123 (76–169)	103 (75–162)	0.436
Insulin (μU/mL)^a^	16.8 (19.9–25.8)	15.7 (10.6–23.9)	0.551
HOMA-IR^a^	3.5 (2.3–5.9)	3.4 (2.4–5.3)	0.912
**Physical activity**			
Exercise (h/wk)^a^	5.5 (3–10)	7 (4–11)	0.051
Sedentary (h/wk)^a^	13 (9.5–18.0)	14 (10.5–20.0)	0.156
**Maternal schooling (%)**			
Primary education or less	14 (16.7)	12 (14.0)	
Secondary	28 (33.3)	39 (45.4)	
High school or more	42 (50.0)	35 (40.7)	0.276

*BMI* Body Mass Index, *HOMA-IR* Homeostasis Model to Assess the Insulin Resistance Index

^a^Median, interquartile range

^b^Pearson *X*^2^ test, *t* Student test for independent data or Mann-Whitney test

Table 3 shows the effect of the educational intervention on children’s dietary habits and behaviors, both within each group and between groups. In terms of dietary habits, intervention group participants showed greater behavioral changes than control group participants.

**Table 3:** Change in children’s eating habits and behavior, by study group

Variables	Intervention (***n*** = 44)					Control (***n*** = 48)					Between groups ***p***^**b**^
	Basal (%)	Final (%)	Points (%)	Relative (%)	***p***^**a**^	Basal (%)	Final (%)	Points (%)	Relative %)	***p***^**a**^	
**Child’s habits at home**											
Breakfast	72.7	84.1	11.4	15.7	0.195	81.3	85.4	4.1	5.0	0.584	0.089
**Habits at lunchtime**											
Putting salt on the table	54.6	27.3	−27.3	−50.0	0.009	62.5	29.2	−33.3	−53.3	0.001	0.752
Putting sugar on the table	50.0	27.3	−22.7	−45.4	0.029	47.9	29.2	−18.7	−39.0	0.059	0.535
Putting sweet soft drinks on the table	47.7	20.5	−27.2	−57.0	0.007	37.5	16.7	−20.8	−55.5	0.022	0.885
Serves the right portion	61.4	93.2	31.8	51.8	< 0.001	61.7	87.2	25.5	41.3	0.005	0.316
Filling the food plate	29.6	4.6	−25.0	−84.5	0.002	31.9	12.8	−19.1	−59.9	0.026	0.009
To serve an additional portion	70.5	40.9	−29.6	−42.0	0.005	72.3	36.2	−36.1	−49.9	< 0.001	0.450
Forcing to finish food	27.3	18.2	−9.1	−33.3	0.309	25.5	23.4	−2.1	−8.2	0.810	0.003
Accepting food substitutions	36.4	20.5	−15.9	−43.7	0.098	52.2	45.7	−6.5	−12.5	0.532	< 0.001
**Frequency of food consumption**											
Fried foods (> 3times/wk)	69.1	57.1	−12.0	−17.4	0.296	71.4	59.5	−11.9	−16.7	0.200	0.932
Roasted foods (daily)	90.1	93.2	3.1	3.4	0.694	82.6	95.7	13.1	15.9	0.041	0.046
Fruits (daily)	69.6	91.3	21.7	31.2	0.009	68.1	72.3	4.2	6.2	0.652	0.002
Vegetables (daily)	50.0	71.7	21.7	43.4	0.033	48.9	55.3	6.4	13.1	0.536	< 0.001
**Frequency of beverage**											
Simple water (daily)	84.4	89.1	4.7	5.6	0.509	91.3	87.0	−4.3	−4.7	0.503	0.310
Sweetened water prepared at home (> 3 times/wk)	80.0	53.3	−26.7	−33.4	0.007	64.4	44.7	−19.7	−30.6	0.057	0.776
Natural juice (> 3 times/wk)	37.2	20.5	−16.7	−44.9	0.084	37.2	18.2	−19.0	−51.1	0.047	0.565
Industrialized juice (> 3 times/wk)	34.1	15.6	−18.5	−54.3	0.043	40.5	20.9	−19.6	−48.4	0.051	0.584
Soft drinks (> 3 times/wk)	84.4	60.0	−24.4	−28.9	0.010	70.5	53.3	−17.2	−24.4	0.097	0.631
**Other habits**											
Time to finish food (< 30 min)	70.5	70.5	0.0	0.0	1.000	75.0	66.7	−8.3	−11.1	0.369	0.023
Children watching TV at mealtime	72.1	46.5	−25.6	−35.5	0.016	57.5	59.6	2.1	3.7	0.834	< 0.001

^a^Pearson *X*^2^ test. ^b^Equal proportions test

### Dietary habits and behaviors at home

The percentage of children who ate breakfast at home improved in both groups without statistically significant differences.

### Habits and behaviors when eating at home

Both groups reduced the habit of placing the salt shaker and soft drinks at the table, and though the intervention group also reduced the habit of putting sugar on the table, there was no difference in these habits between groups. Regarding dietary habits, both groups improved their portion sizes and serving habits, with no difference between groups. Both groups improved the way they placed food their plate though the intervention group showed greater improvement (*p* = 0.009). The habit of returning for seconds decreased significantly in both groups, with no difference between them. As far as forcing children to finish servings and allowing them to substitute food, there was a significant difference between groups favoring the intervention group, (*p* = 0.003), (*p* < 0.001), respectively.

### Food consumption

Consumption of grilled or shallow-fried foods increased in the control group (*p* = 0.046), which means cooking meat and/or vegetables directly on a hot plate with minimal or no added fat; while the intervention group improved their daily consumption of fruits (*p* = 0.002) and vegetables (*p* < 0.001).

### Beverage consumption

Water intake showed no significant changes. However, intervention group participants reduced their consumption of industrially-produced juice and soft drinks, as well of sweetened water. In Mexico these beverages are prepared by blending fresh fruit, water, and sugar; in this category are included lemonades and water made by boiling hibiscus flowers. Control group consumption of natural fruit juice decreased; the differences between groups were not significant.

### Other dietary habits

Duration of eating time improved in the control group (*p* = 0.023), while the habit of eating in front of the television, decreased (*p* < 0.001) in the intervention group.

Table 4 shows changes in anthropometric and metabolic in children during the follow-up. No effect was found on anthropometric measurements in the intervention group. However, in the metabolic parameters a decrease was found in insulin concentrations (− 3.7; 95% CI − 6.8 to − 0.67) and in HOMA-IR levels (− 0.89; 95% CI 1.69 to − 0.9) in the intervention group.

**Table 4:** Change in anthropometric and metabolic parameters in children during the follow-up

	Intervention effect, β (95% CI)	***p***^**a**^
**Anthropometrics**		
BMI (percentile)	−0.26 (−0.97 to 0.46)	0.488
Waist circumference (cm)	0.19 (−1.0 to 1.41)	0.751
**Metabolics**		
C-HDL (mg/dL)	−0.6 (−3.6 to 2.4)	0.695
Triglycerides (mg/dL)	−9.9 (−25.8 to 5.8)	0.216
Glucose (mg/dL)	1.9 (−2.9 to 6.7)	0.446
Insulin (μU/mL)	−3.7 (−6.8 to −0.67)	0.017
HOMA-IR	−0.89 (1.69 to − 0.9)	0.029

*BMI* Body Mass Index, *HOMA-IR* Homeostasis Model to Assess the Insulin Resistance Index

^a^Linear regression mixed effects models, adjust for baseline data of the dependent variable, time, baseline age, gender, maternal schooling and baseline BMI percentile

**Fig. 2:**
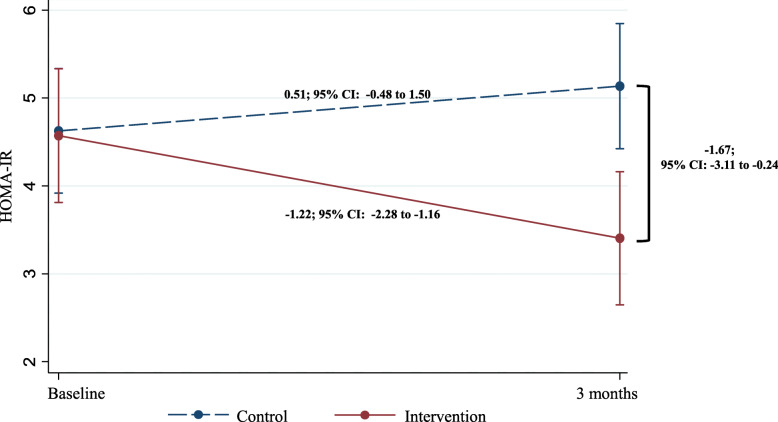
Changes in HOMA-IR levels in children during follow-up. Linear regression mixed effects model with marginal analysis, mean HOMA-IR by group and by time, adjust for baseline data of the dependent variable, visit, baseline age, gender, maternal schooling and baseline BMI percentile

On the basis of a linear regression model with mixed effect, the interaction between the groups and the evaluation time of the dependent variable HOMA-IR were evaluated. Mean HOMA-IR by group (intervention or control) and by time was calculated and a graphic was done using marginal analysis. In Fig. 2, the dashed line shows that children in the control group slightly increased HOMA-IR levels (0.51; 95% CI − 0.48 to 1.50), while the solid line shows that children in the intervention group significantly decreased HOMA-IR levels (− 1.22; 95% CI − 2.28 to − 1.16). The difference in HOMA-IR between the control and intervention group at the end of the follow-up was − 1.67; 95% CI: − 3.11 to − 0.24.

## Discussion

Today, people of all ages around the globe are immersed in a culture that intakes more energy than it can use. Mass media encourage children to eat foods that are dense in saturated fat, refined sugars, and salt [27]. Meanwhile, educational and health institutions promote healthy dietary habits and physical activity but fail to counteract the culture of unhealthy food consumption. The result is an obesity epidemic that has coined the term “Globesity” in an attempt to address both the causes of this epidemic and its severe and onerous consequences [28].

In this study, HOMA-IR changes were observed in obese children belonging to the intervention group, this may be associated with a change in children’s nutritional status but mainly by changes in eating habits. Likewise, it has been observed that people who are obese and reduce their body weight improve their metabolic biomarkers and increase their insulin sensitivity [29, 30]. Programs encouraging healthy eating should be promoted among the population as a way of improving lipid profiles and preventing or reverting insulin resistance, as well as preventing chronic disease.

In the present, more than a third of children in Mexico of ages 5–11 and adolescents 12–19 are overweight and obese [9]. Hypothetically speaking, if they were all to seek help for their problem from health services, these institutions would be unable to cope. On the other hand, conditions are so limited that health workers often issue parents diets for their children and physical activity recommendations the way they would prescribe medication. This medical-style approach has not had the expected results. Modifying habits, in this case dietary habits, requires an educational process that involves mothers because they are largely in charge of household food practices [31, 32]. This educational intervention, where the mothers of obese children participated in a group, took place in a clinical setting, in a hospital, and not in homes. However, its messages sought to modify daily or routine feeding behaviors at home, in the microsystem where obese children reside according to Bronfenbrenner’s ecological model [33], as opposed to having to prepare a menu as prescribed in the usual nutritional consultation, to which children would have to adhere to improve their health. The priority during education sessions was for mothers to identify behaviors that promoted greater energy intake either by quantity, density or frequency, to then modify their children’s dietary habits and improve their nutritional status. The content provided at the sessions did not include information or activities for increasing physical activities that would promote energy output.

By the end of the study, there was an improvement in intervention group feeding behaviors, such as reducing portion sizes, avoiding full plates, not forcing children to finish their meals and accepting food substitutions. Both groups became less prone to putting salt and bottled soft drinks on the table, and reduced the habit of repeating servings, but without difference between groups. In terms of food consumption, the intervention group participants increased fruit and vegetable intake in their diets, a change that is key within the healthy diet model [19], while control group participants increased their consumption of grilled or shallow-fried foods.

Regarding beverages, intervention group participants decreased their intake of domestic and industrially-produced sweetened beverages, while control group participants only reduced their consumption of natural juice. A note worthy observation is that neither group modified their water intake, which suggests that the information given to families highlighted the risk of sweetened drinks to the health of obese children more than the benefit of simple water intake. Other studies conducted on school-aged children and their parents addressing a range of components including greater fruit, vegetable, and water intake have had positive impacts on children [34, 35].

In other noted behaviors, control group children became less prone to finishing their meal in under 30 min; the occurrence of intervention group children watching television at mealtimes decreased, which, as is known, encourages them to taste their food without distractions or subliminal messages from advertisers [27, 36].

After 3 months, aside from a decrease in BMI percentile that registered no difference between groups, changes in eating behaviors did not improve anthropometric indicators in children from either group. In a study conducted in a school setting in which parents and children attended 15 educational sessions promoting healthy eating and physical activity, as opposed to another group that only attended two sessions, participants in the group with the highest number of contacts improved their anthropometric indicators [37]. Other studies on children ages 4–12 that were conducted in homes over a 1 year-period achieved minimal changes in diet, physical activity, and body weight [38].

Among the weaknesses encountered during this study, the high desertion rate is the most relevant. Holding face-to-face sessions for mothers who live in large cities such as Mexico City can be complicated by distance, prolonged travel times, and transport costs. Another important consideration is the reduced size and scarcity of spaces for educational activities within health institutions. For these reasons, technological alternatives such as the internet and mobile phones must be explored as means of sending users relevant information and as a way of eliminating the need for mothers to travel to health clinics [39]. Also, parents who are less participative in these duties may become more engaged [40]. Another important consideration is that follow-up sessions and duration of contact between researchers and mothers was not enough to achieve the desired anthropometric objectives [17].

In regard to strengths, behavioral changes in intervention groupchildren surrounding meals and food preparation can be considered relevant, along with a significant HOMA-IR index decrease which was significant between groups. Metabolic profiles in obese children have been found to improve with physical activity interventions that do not include dietary provisions [41]. In this study, which did not address physical activity, we found that modifying dietary habits can also achieve these benefits, possibly in a longer-lasting way.

Involving the mothers of obese children in group sessions where they are given information on how to modify dietary habits in the home microsystem leads to beneficial health changes in their children, though more time is required to achieve effects on anthropometric measurements.

## Conclusion

The intervention aimed at children suffering from obesity and their mothers modified some dietary habits and behaviors at home and improved insulin levels and HOMA-IR in a context where they were not given a specific diet as a treatment nor did they receive intervention on physical activity. Therefore, group sessions aimed at the mother to modify dietary habits at home can be an extra resource in the management of childhood obesity in the health institutions.

## Supplementary information

**Additional file 1.** CONSORT 2010 checklist of information to include when reporting a randomised trial

## Data Availability

The datasets used and/or analyzed during the current study are available from the corresponding author on reasonable request.

## Abbreviations

BMI: Body Mass Index
HOMA-IR: Homeostasis Model to Assess the Insulin Resistance Index
SSB: Sugar Sweetened Beverages
